# Refractory Chylothorax and Ventricular Hypertrophy Treated with Trametinib in a Patient with Noonan Syndrome: 18-Month Follow-Up

**DOI:** 10.3390/children11111342

**Published:** 2024-10-31

**Authors:** Antonia Pascarella, Giuseppe Limongelli, Alessandro De Falco, Elia Marco Paolo Minale, Giangiacomo Di Nardo, Giovanni Maria Di Marco, Geremia Zito Marinosci, Giorgia Olimpico, Paolo Siani, Daniele De Brasi

**Affiliations:** 1 Unit of Chronic and Multifactorial Diseases, Santobono-Pausilipon Children’s Hospital, 80129 Naples, Italy; a.pascarella@santobonopausilipon.it (A.P.); p.siani@santobonopausilipon.it (P.S.); 2 Inherited and Rare Cardiovascular Diseases Unit, Department of Translational Medical Sciences, University of Campania “Luigi Vanvitelli”, Monaldi Hospital, 81031 Naples, Italy; 3 U.O.C. Genetica Medica, A.O.U. Federico II, 80131 Naples, Italy; e.minale@studenti.unina.it; 4 Department of Pediatric Cardiology, Santobono-Pausilipon Children’s Hospital, 80129 Naples, Italy; gg.dinardo@libero.it (G.D.N.);; 5 Pediatric ICU, Santobono-Pausilipon Children’s Hospital, 80129 Naples, Italy; 6 Department of Translational Medical Science, Section of Pediatrics, University “Federico II”, 80131 Naples, Italy; giorgiaolimpico@libero.it; 7 Medical Genetics Unit, Santobono-Pausilipon Children’s Hospital, 80129 Naples, Italy; d.debrasi@santobonopausilipon.it

**Keywords:** Noonan syndrome, targeted molecular therapy, MEK inhibitor, hypertrophic cardiomyopathy, pulmonary stenosis, refractory chylothorax

## Abstract

RASopathies are a group of genetic syndromes caused by germline mutations in genes involved in the RAS/Mitogen-Activated Protein Kinase signaling pathway, which regulates cellular proliferation, differentiation, and angiogenesis. Despite their involvement at different levels of this pathway, RASopathies share overlapping clinical phenotypes. Noonan syndrome is the most prevalent RASopathy, with an estimated incidence of 1 in 2500 live births, and it is typically inherited in an autosomal dominant manner, with 50% of cases involving gain-of-function mutations in the PTPN11 gene. De novo mutations are common, accounting for 60% of cases. The phenotype of Noonan syndrome includes characteristic facial and physical features, congenital cardiac defects, lymphatic and cerebrovascular anomalies, renal malformations, hematological abnormalities, developmental issues, and an increased risk of cancer. Severe congenital cardiac defects and lymphatic abnormalities significantly impact prognosis, contributing to increased morbidity and mortality. Recent therapeutic advancements have introduced trametinib, an MEK1/2 inhibitor, for treating Noonan syndrome patients with severe cardiac and lymphatic complications. To assess its efficacy, here, we present a case of a newborn with Noonan syndrome who exhibited refractory chylothorax, ventricular hypertrophy, and pulmonary stenosis who was treated with trametinib. The patient demonstrated significant improvement in chylothorax and left ventricular hypertrophy, though pulmonary stenosis persisted. This case further confirms trametinib’s potential as a therapeutic option for severe Noonan syndrome complications, emphasizing the need for further clinical trials to optimize treatment protocols and evaluate long-term outcomes.

## 1. Introduction

RASopathies are a group of genetic syndromes caused by germline mutations in one of the genes involved in the RAS/Mitogen-Activated Protein Kinase (MAPK) signaling, a regulatory pathway associated with cellular proliferation, differentiation, and angiogenesis. These disorders, including neurofibromatosis type 1, Noonan syndrome, cardiofaciocutaneous syndrome, Costello syndrome, and Legius syndrome, present overlapping clinical phenotypes because the same signaling pathway is altered although at different levels. Particularly, Noonan syndrome (NS) is the most common RASopathies with an estimated prevalence of 1 in 2500 among live births [1], and it is the second most common syndromic cause of congenital heart disease after trisomy 21 [2]. To date, germline mutations in thirteen genes involved in the RAS/MAPK pathway have been identified in NS and, in most cases, the inheritance manner is autosomal dominant [3]: 50% of described cases present a gain of function mutation in the *PTNP11* (protein tyrosine phosphatase non-receptor type 11) gene [4,5]. De novo mutations are common, accounting for 60% of cases [6].

The phenotype of NS is characterized by a variable expression so that we can define a phenotypic spectrum, including distinctive facial features (wide-set eyes, low-set ears, vivid blue or blue-green irises, epicanthal folds, ptosis), physical features (webbed neck postnatal short stature, chest and skeletal malformations), organ abnormalities (congenital cardiac defects, lymphatic and cerebrovascular anomalies, renal malformation), neuropsychological features (sensorineural hearing loss, developmental or behavioral problems), other abnormalities (cryptorchidism, abnormal skin pigmentation, coagulation defects, feeding difficulties), and an increased risk of cancer [7,8].

Congenital cardiac defects, such as pulmonary stenosis and hypertrophic cardiomyopathy (HCM), and many prenatal and/or postnatal lymphatic abnormalities, are often severe and can influence the prognosis of disease, increasing morbidity and mortality [9,10,11,12].

Lymphatic dysplasia prenatal ultrasound often shows polyhydramnios, increased nuchal translucency, cystic hygroma, non-immune hydrops fetalis, or pleural effusion [13]. Prevalence data of postnatal lymphatic anomalies are still lacking, but it is estimated at around 20%, although it could be higher [14]. Postnatal lymphatic abnormalities can occur in infancy, childhood, or adulthood, and the most common ones are peripheral lymphoedema, pulmonary, testicular or intestinal lymphangiectasia, chylous effusions of the pleural space and peritoneum, pericardial effusion, hypoplastic leg lymphatic vessels, anomalous thoracic cage lymphatic vessels, aplasia of the thoracic duct, hypoplastic inguinal and iliac lymphatic vessels, and lymphoedema of the scrotum or vulva [15,16]. Swarts and colleagues [17] have conducted a retrospective cohort study which shows no correlation between prenatal/postnatal lymphatic anomalies and congenital heart disease, although pulmonary lymphatic abnormalities may worsen cardiac outcome; moreover, there is no clear genotype/phenotype association regarding lymphatic features, especially in low-prevalent genes.

Trametinib is a highly selective reversible allosteric inhibitor of MEK1/2 activity, approved in the treatment of cancers with the activation of the RAS/MAPK pathway, such as BRAF V600E mutated metastatic melanoma and BRAF V600E mutated non-small-cell lung cancer [18,19]. In 2019, Andelfinger and colleagues were the first to use trametinib in two patients with NS caused by RIT1 mutations and severe early-onset HCM; the treatment resulted in the resolution of the cardiac hypertrophy after three months of therapy [20]. Subsequently, trametinib was administered to other NS patients with different genotypes affected by HCM and/or severe lymphatic abnormalities [21,22,23,24,25,26,27,28] and multifocal atrial tachycardia [29,30] with encouraging results.

Herein, we describe the management of refractory chylothorax, ventricular hypertrophy, and pulmonary stenosis in a newborn patient affected by NS and treated with trametinib.

## 2. Materials and Methods

**Figure 1:**
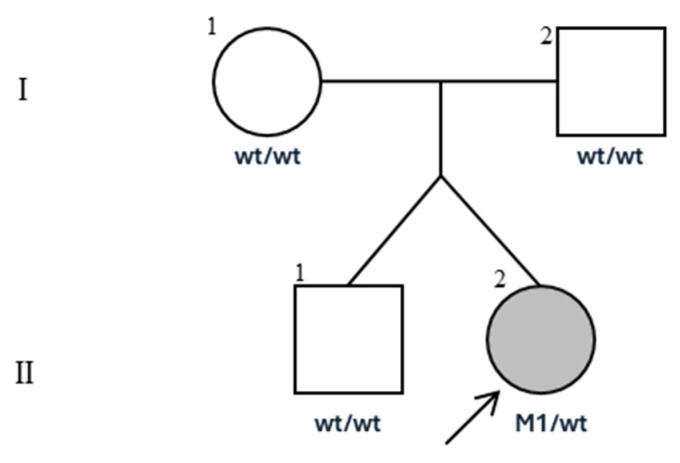
Pedigree chart of the proband. Wt: wild type; M1: p.Phe285Ser variant in the *PTPN11* gene.

We report a case of a female patient who is the second of two twins of healthy non-consanguineous parents (Figure 1). She was born late preterm at 37 weeks of gestation age by urgent cesarean delivery for fetal distress detected by cardiotocography. Gestation was complicated by diamniotic dichorionic twin pregnancy with gestational diabetes and evidence of polyhydramnios in the third trimester. Her birth weight was 2900 g (appropriate for gestational age, 50–75 percentiles), length 46 cm (25 percentile), occipital frontal circumference (OFC) 35 cm (90–97 percentiles), and the Apgar score was 4–6–7 at 1, 5, and 10 min, respectively.

At birth she was pale, hypotonic with hyporeflexia, and had a heart rate <100 bpm. Cardiopulmonary resuscitation (CRP) was performed, and the patient required urgent intubation and invasive mechanical ventilation. The arterial blood gasses performed at 30 min of life showed a pH of 7 with a base deficit of –15 and a lactate of 10 mmol/L. Based on the blood gasses, Apgar scores, and modified Sarnat neurologic assessment, hypoxic–ischemic encephalopathy (HIE) was diagnosed. Therapeutic hypothermia was performed and maintained for 72 h.

At birth, the patient presented the following phenotypic features: ptosis, down-slanted palpebral fissures, marked webbed neck, epicanthic folds, low-set posteriorly rotated ears, and prominent frontal bossing.

M-mode Doppler echocardiography, performed in the first 24 h after birth, showed mild hypertrophy of the left ventricle with increased thickness of the end diastolic left ventricular posterior wall (LVPWd: 4.6 mm, Z-score 2.8), right ventricular hypertrophy with hypertrabeculation, normal values of interventricular septum in diastole (SIVd: 4.2 mm, Z-score: 1.17), and tricuspid annular plane systolic excursion (TAPSE: 16 mm); dysplastic pulmonary valve with moderate supravalvular stenosis with an estimated transvalvular maximum pressure gradient of 40 mmHg and ostium secundum atrial septal defect with mild left-to-right shunt were also detected. A left ventricular ejection fraction (LV-EF) was preserved, and nt-pro-BNP and cardiac Troponin T (cTnT) were normal. Propranolol (1 mg/kg/day) was introduced.

**Figure 2:**
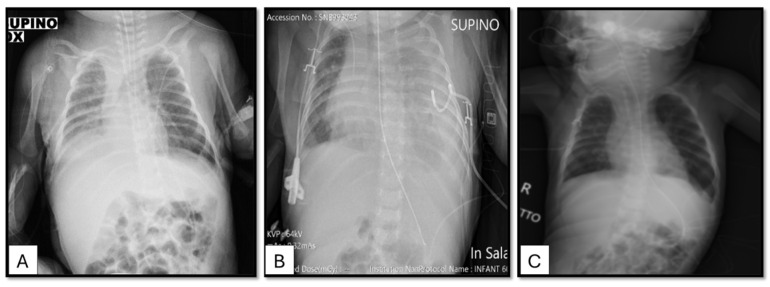
Evaluation of chylothorax of the proband by chest X-rays before and after treatment with trametinib. (**A**) Patient on the second day of life: right pleural effusion. (**B**) Patient after octreotide therapy: worsening of the pleural effusion. (**C**) Patient after 24 days of trametinib therapy: absence of chylothorax.

On the second day of life, lung scan ultrasound showed right pleural effusion (maximum diameter of 13 mm) and parenchymal lung consolidation mostly involving the basal field of the right lung, which was also confirmed by chest radiography (Figure 2A).

In the following days, as the pleural effusion doubled in size (maximum diameter of 26 mm) and the respiratory dynamics deteriorated, thoracic drainage was positioned on the tenth day of life. The drained fluid appeared milky, and the laboratory analysis demonstrated high lymphocyte (42,080 cell/mmc) and triglyceride concentrations (167 mg/dL); all these characteristics were suggestive of chylothorax.

Clinical features, including chylous effusion, cardiac abnormalities, and phenotypical features, suggested a genetic alteration in the RAS/MAPK pathway, so a next-generation sequencing-targeted RASopathies panel, including the coding regions and the intronic regions near splice sites of 23 genes (*A2ML1*, *AKT3*, *BRAF*, *CBL*, *CCND2*, *HRAS*, *KRAS*, *MAP2K1*, *MAP2K2*, *NF1*, *NRAS*, *PIK3CA*, *PIK3R2*, *PTPN11*, *RAF1*, *RASA1*, *RASA2*, *RIT1*, *RRAS*, *SHOC2*, *SOS1*, *SPRED1*, *STAMBP*), was performed, revealing the c.854T>C, p.Phe285Ser variant in heterozygosis in the *PTPN11* gene (NM_002834.4). The variant is cataloged in the ClinVar database with the accession number rs121918463, and it is classified as a pathogenic variant according to the American College of Medical Genetics and Genomics (criteria PS3, PP3, PS2, PM2, PM5, PP2) [31]. Parental NGS testing revealed that the mutation arose de novo, and it was absent in her twin brother, who was asymptomatic (Figure 1).

Following right pleural drainage, continuous octreotide intravenous infusion (6 µg/kg/h) and intravenous furosemide (2 mg/Kg/day) were started in association with medium-chain triglyceride (MCT) parenteral nutrition. After 38 days, a partial bettering of pleural effusions was achieved and respiratory function bettered so she was extubated and started non-invasive ventilatory support with continuous positive airway pressure (nCPAP) and progressively reduced and stopped octreotide treatment. On the sixty-three day of life was reached, the patient presented dyspnea with hypercapnia, and chest ultrasound and X-ray examinations showed relapse of left pleural effusion. Therefore, octreotide intravenous therapy was reintroduced at the maximum dosage (10/kg/h) in addition to pleural drainage. Despite this treatment, the patient developed bilateral pleural effusion (Figure 2B) with progressive decline in respiratory function and a need for nCPAP, high flow nasal cannula (HFNC), alongside repeated therapeutic thoracentesis.

## 3. Off-Label Trametinib Treatment for Chylothorax

Due to the failure of the first-line treatment for chylothorax, trametinib was introduced as an off-label prescription after obtaining parent informed consent. Trametinib was administered orally at a starting dose of 0.025 mg/kg daily on the 118th day of life.

**Figure 3:**
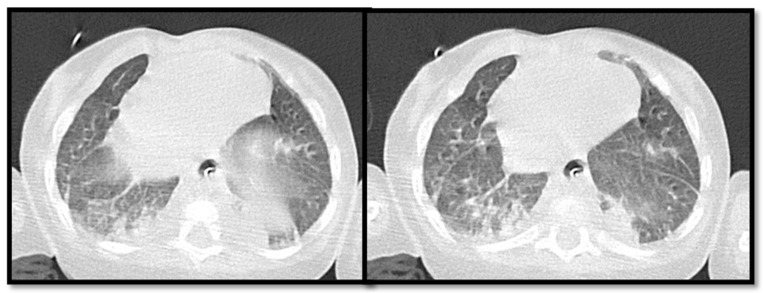
Pulmonary high-resolution CT scan (HRCT) performed after 14 days of the beginning of trametinib therapy. There are multiple parenchymal lung consolidations associated with diffuse ground glass opacities.

After 14 days of trametinib therapy, pulmonary high-resolution CT scan (HRCT) showed parenchymal lung consolidation involving the anterior segment of the upper lobe, the medial segment of the middle lobe, and the medial basal and posterior basal segments of the right lower lobe associated with diffuse ground glass opacities (Figure 3).

After 24 days of the beginning of the therapy, no pleural effusion on chest ultrasounds and X-rays were found, and the respiratory dynamics improved until weaning from the non-invasive mechanical ventilation (Figure 2C).

On the other hand, M-mode Doppler echocardiography on the 121th day of life showed worsening of pulmonary stenosis with an estimated transvalvular gradient (TG) of 105 mmHg. The patient underwent percutaneous balloon pulmonary valvuloplasty (PBPV) with partial efficacy: after the procedure, the maximum TG was 65 mmHg.

**Figure 4:**
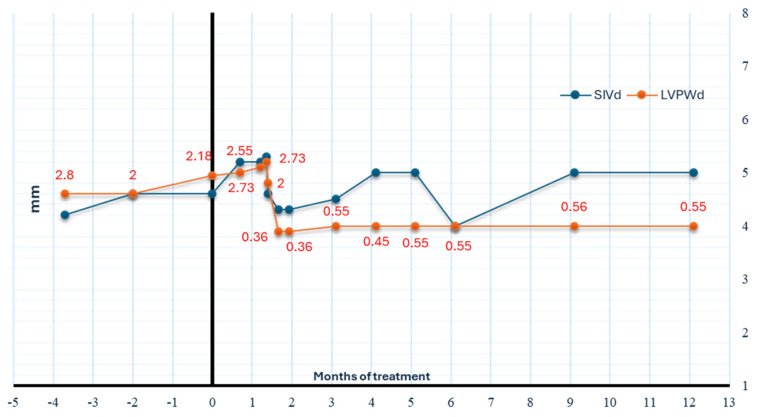
Graph displaying improvement in the thickness of the posterior wall of the left ventricular in relation to the beginning of trametinib therapy. The measurements of the interventricular septum (SIVd), assessed by M-Mode Doppler echocardiography, remain stable. Regarding the LVPWd, body surface area and weight-adjusted z-score of M-mode measurements have been reported (http://www.parameterz.com/refs/kampmann-heart-2000, accessed on 10 October 2024). SIVd: interventricular septum, end diastole, LVPWd: left ventricular posterior wall, end diastole.

The 12-month follow-up showed improvement in the left ventricular size, which returned to the normal range (Figure 4). The 18-month follow-up showed no recurrence of the chylothorax assessed by chest ultrasound.

However, right ventricular hypertrophy did not improve after the pulmonary valvuloplasty; tricuspid annular plane systolic excursion (TAPSE), which assess the functionality of the right ventricle, showed values between 12 and 16 mm. The trend of the pulmonary gradient remained stable with a maximum value of 65 mmHg. The treatment with trametinib did not result in an improvement in the pulmonary stenosis, and even after the dilation intervention, the transvalvular gradient remained high. Due to the residual right ventricular hypertrophy and pulmonary stenosis, the patient required treatment with propranolol and furosemide, albeit at low doses. At the 12-month follow-up, the patient was being treated with propranolol (1.5 mg/Kg/day) and furosemide (0.5 mg/kg/day).

After 32 days of therapy with trametinib, the patient presented eczema on the scalp and face, without other relevant side effects. After 2 months of trametinib, we detected a reduction in LVPWd (3.9 mm, z score 0.36).

At 20 months of age, her weight was 7980 kg (3–10 percentiles according to NS-specific growth charts) and length 78 cm (3–10 percentiles NS-specific growth charts).

## 4. Discussion

Up to recent years, the only therapeutic options for patients for NS with serious comorbidities (such as HCM and severe lymphatic abnormalities) were limited to surgery, symptomatic pharmacological treatment, and heart transplantation [32]. In particular, regarding chylothorax, the treatments currently available in pediatric patients, including some used in patients with Noonan syndrome, are low-fat diet with the addition of medium-chain triglycerides (MCTs), total parenteral nutrition (TPN), and pleural drainage. If there is no response or in refractory cases, somatostatin or its analogs (such as octreotide) can be considered [33]. Sirolimus has also been used with encouraging results in refractory chylothorax cases, especially when associated with complex lymphatic anomalies [34]. Surgical approaches, such as chemical or surgical pleurodesis, thoracic duct embolization, or repair, are considered in severe or persistent cases depending on the etiology. The experience with Midodrine, an oral alpha-1-adrenoreceptor agonist, in pediatric patients is limited, but it has been used with good results in a neonate with trisomy 21 complicated by lymphatic dysplasia [35].

Thanks to the spread of preclinical studies on targeted agents specific for the RAS/MAPK pathway, new therapeutic strategies have become available [36]. In 2020, selumetinib, an MEK inhibitor, was approved in patients with inoperable plexiform neurofibromas affected by neurofibromatosis type 1 (NF1) with hyperactivation of the RAS-MAPK signaling pathway [37]. Recent encouraging results of a phase 2 study have been published regarding the use of trametinib in pediatric NF 1 patients with refractory glioma and plexiform neurofibroma and MAPK/ERK pathway activation [38]. Furthermore, trametinib has recently been used as an off-label drug in NS pediatric patients with a well-characterized germline pathogenic variant, affected by cardiac disease and/or lymphatic anomalies and cardiac arrhythmia and showing overall encouraging results, especially in relation to lymphatic anomalies (Table 1). Eligibility for treatment with trametinib in NS is currently limited to a subset of patients in life-threatening and rapidly progressive clinical conditions that are unresponsive to the first-line treatment or in the absence of therapeutic alternatives (progressive HCM, severe lymphovascular disease, heart arrhythmias). Trametinib is a second-generation small-molecule inhibitor of MEK kinase. It functions as an allosteric, ATP noncompetitive inhibitor with activity against both MEK 1 and MEK 2 kinases. The inhibitory effect of trametinib on cell growth was shown to be through the inhibition of p-ERK ½, which specifically prevents RAF-dependent MEK phosphorylation and prolongs the inhibition of phosphorylated ERK (a substrate of MEK) [39].

Our patient, with typical clinical features worsening by hypoxic–ischemic encephalopathy, harbors the missense p.Phe285Ser variant in heterozygosis in PTPN11 gene, encoding for the tyrosine phosphatase protein, non-receptor type 11 (SHP2), which functions as a positive regulator of the RAS/MAPK pathway by integrating signals from growth factors [40]. The p.Phe285Ser variant is one of the most common mutation at position 285 in the *PTPN11* gene and has been associated with NS patients with chylothorax [41].

The patient’s refractory chylothorax responded quickly at the beginning of therapy with trametinib (approximately 10 days). The subsequent follow-up confirmed the temporal pattern of the response to the MEK 1–2 inhibitor, as described in the literature before [32].

In our patient, worsening of the pulmonary stenosis was observed and valvuloplasty was necessary, although it was partially effective. The continued use of trametinib unfortunately did not improve the degree of pulmonary stenosis. Improvement in the right ventricular outflow tract obstruction (RVOT) gradient after two months of treatment, before percutaneous balloon dilatation of the valve, has been described in an NS patient with a pathogenic *RIT1* variant, pulmonary valve stenosis, and HCM [25]. It cannot be ruled out that trametinib non-responsive pulmonary stenosis in our patient reduced the efficacy of treatment with trametinib on right ventricular hypertrophy. The effectiveness of the trametinib may be reduced by some concurrent clinical conditions as it was not effective on pulmonary vascular disease and severe pulmonary arterial hypertension, as described in a premature newborn [22]. Trametinib is not ineffective in all the cases (Table 1) for improving right heart function and pulmonary hypertension in NS. In *RIT1* mutations, it either improves or does not worsen (cases 1, 2, 8, and 12 in Table 1), while in *RAF1* mutations, there are cases where it is effective (case 4) and cases where it is ineffective (case 5). Case 10, which has the same genetic mutation as our patient, is also described as ineffective. Different genetic mutations within the genes responsible for the RASopathies can variably influence the RAS-MAPK pathway and lead to different clinical manifestations. In fact, patients with *RIT1* mutations may have a variable response to trametinib, ranging from improvements to no worsening; this may depend on the role of *RIT1* in the pathway, where some mutations specifically affect the heart and pulmonary vasculature. Patients with *RAF1* mutations tend to have more pronounced cardiac hypertrophy. In some cases, trametinib may be effective, while in others it is ineffective, possibly due to the variability in the ability of these mutations to hyperactivate the MEK-ERK pathway, which trametinib aims to inhibit. Hypertrophic cardiomyopathy associated with Noonan syndrome can respond differently depending on how mutations affect the activity of the RAS-MAPK pathway. Mutations located higher or lower in the pathway may differently alter sensitivity to trametinib, which acts at a specific stage of the cascade. In summary, the variable effect of trametinib in NS patients is due to the complexity of the RAS-MAPK pathway and the specificity with which each mutation influences signaling at the cardiac level. Individual differences in the pathophysiology caused by each mutation thus determine a variability in cardiac response to this treatment.

Uncertainty on the duration of treatment with trametinib was partially clarified by Bruce D. Gelb and collaborators [32]. After two years of treatment with trametinib in two patients with HCM, an attempt to discontinue the drug failed as increases in NT-pro-BNP and wall thicknesses were observed in both patients. Trametinib was then used for another year, and subsequent discontinuation showed no relapse in the 2-year follow-up. However, a variable response to treatment between patients and consequently the need for a shorter or longer treatment duration cannot be excluded.

The starting dosage of trametinib in NS pediatric patients reported in the literature is 0.02–0.027 mg/kg, given orally once daily [42]. Different dosages are currently used in pediatric refractory tumors characterized by the activation of the MAPK/ERK pathway as low-grade gliomas: 0.032 mg/kg once daily for patients age < 6 years and 0.025 mg/kg once daily for patients age ≥ 6 years [43]. In a study on the safety, pharmacokinetics, pharmacodynamics, and efficacy for the dose-escalation of trametinib used in adult patients with solid tumors, the reported side effects were skin effects, diarrhea, fatigue, peripheral/periorbital edema, nausea, vomiting, pruritus, dry skin, chapped skin or skin fissures, decrease appetite, ocular toxic effects (including retinopathy, glaucoma, photophobia, reduced visual acuity, retinal hemorrhage), mucosal inflammation, constipation, left-ventricular dysfunction, thrombocytopenia, and dry mouth [39]. In pediatric cancer patients, trametinib is often used in co-administration with other drugs; the most commonly reported adverse effects are diarrhea, constipation, acneiform rash, retinopathy, uveitis, interstitial lung disease, hemorrhage, venous thromboembolism, and hypertension [44]. Our patient experienced only transient eczema that did not require specific treatments. To date, no serious adverse events have been reported in either our patient or in NS pediatric patients in the literature treated with trametinib. Neither the long-term tolerability and safety of Trametinib nor its effectiveness for other clinical manifestations are currently available for individuals with NS.

To follow-up thorax lymphatic anomalies in our patient, we performed sequential chest ultrasound rather than chest CT or chest radiography because of its simplicity and accuracy and to avoid excessive radiation exposure. Recently, Nakano T.A. et al. [24] used a surveillance protocol for monitoring known side effects based on its use in adults with cancer who do not have germline over the activation of RAS; they also used a protocol to monitor the outcome measures with instrumental tests especially to evaluate the improvement in the chylothorax and lymphatic anomalies. In particular, in a 12-month follow-up of three patients affected by lymphatic anomalies to monitor the results obtained with trametinib, chest X-rays were performed after 2, 4, 24, and 48 weeks of treatment, chest/abdominal MRI or CT after 24 and 48 weeks of treatment, and DC-MRL (dynamic contrast magnetic resonance lymphangiography) after 48 weeks of treatment. All three patients had DC-MRL evidence of primary central lymphatic dysplasia before starting treatment with trametinib. It would be desirable to use shared and standardized surveillance protocols as well as the measurement of outcomes, especially for longer follow-up.

## 5. Conclusions

Our case and the published cases confirm the efficacy of treatment with trametinib in specific clinical conditions in patients affected by NS. Further clinical experiences may facilitate clarifying the therapeutic response in the wide genotypic heterogeneity of other RASopathies and how some comorbidities can modify or influence the success of the treatment.

Long follow-up studies would be useful to both evaluate the possibility of suspending treatment after achieving results and to assess the timing of any relapses; this could lead to evaluating the discontinuation of treatment over time. Furthermore, long-term monitoring of treated patients would provide more information regarding the safety of the treatment. Despite the few cases reported in the literature that show encouraging results, it cannot be excluded that any negative results may depend on the severity of the clinical features at the time of the beginning of therapy.

The use of trametinib has certainly changed the outcomes for serious conditions such as congenital hypertrophic cardiomyopathy and lymphatic abnormalities in patients with NS, conditions previously associated with high mortality. Therefore, it is essential to establish guidelines or recommendations for using trametinib in patients with Noonan syndrome; these guidelines would help navigate clinical situations, especially in critical or life-threatening cases where trametinib could serve as a first-line therapeutic option. Having a clear direction can make a difference in ensuring that patients receive the best possible care in a timely manner.

## Figures and Tables

**Table 1:** Use of trametinib in NS patients: review of the literature.

Mutations	Heart Disease/ Arrhythmia	Lymphatic Abnormalities/ DCMRL	Other Clinical Features	ABT	Dose	Duration of Treatment	Results	Adverse Events	References
*RIT1* (NM_006912.5): c.104G>C, p.Ser35Thr (het, d.n.)	HCM, SBS	Not reported	Prenatal dysplasia of all four valves, polyhydramnios, postnatal macrosomia, hypertelorism, and low-set ears.	14 wks	0.02 mg/kg/day	17 mo	Regression of HCM, improvement SBS, bettering of growth.	None	Andelfinger et al., 2019 [20]
*RIT1* (NM_006912.5): c.246T>G, p.Phe82Leu (het, d.n.)	Progressive biventricular HCM, SBS, pulmonary congestion with postcapillary pulmonary hypertension.	Bilateral chylothorax/ Not performed	Polyhydramnios	13 wks	0.02 mg/kg/day	17 mo	Regression of HCM, improvement of SBS, regression of pulmonary edema, resolution of chylous effusions, bettering of growth.	None	Andelfinger et al., 2019 [20]
*SOS1* (NM_005633): c.2536G>A, p.Glu846Lys (het)	Not reported	Persistent left chylothorax, protein-losing enteropathy with hypoalbuminemia, anemia, abnormal electrolytes levels/Diffusely abnormal central lymphatic system with retrograde mesenteric flow. Extensive perfusion of the left chest and lung.	Difficulty in gaining weight, chronic fatigue, Hashimoto thyroiditis, delayed puberty, growth hormone deficiency, Attention-deficit/ hyperactivity disorder.	15 y	0.01 mg/kg per dose (0.5 mg daily) for tolerability for 1 week; then 1 mg/day	6 mo	Disappearance of the left sided pulmonary interstitial and intercostal lymphatic networks, reduction in retrograde mesenteric flow and resolution of the lymphatic leaks, bettering of growth	None	Dori et al., 2020 [21]
*RAF1* (NM_002880.4): c.770C>T, p.Ser257Leu (het, d.n.)	Biventricular HCM, pulmonary valve stenosis atrial septal defect/ MAT, fibrillation and polymorphic ventricular tachycardia	Chylothorax/not performed	Not reported	20 wks	0.025 mg/kg/day	6 mo	Resolution of MAT, Improvement of biventricular HCM	None	Meisner et al., 2021 [29]
*RAF1* (NM_002880.4): c.770C>T, p.Ser257Leu (het, d.n.)	Severe biventricular obstructive HCM, dysplastic pulmonary valve, pulmonary hypertension	Not reported	Cerebral ventricular hemorrhage (Grade II) with post-hemorrhagic hydrocephalus	47th day of life	0.022 mg/kg/day	Death on day + 57.	Initially improvement in the overall clinical conditions, reduction in the septal thickness and nt-proBNP. Finally developed pulmonary hypertension and severe congestive heart failure	None	Mussa et al., 2021 [22]
*BRAF* (NM_004333.6): c.770A>G, p.Gln257Arg (het)	HCM	Genital and bilateral lower limb lymphoedema, bilateral refractory chylothorax, intestinal lymphangiectasia/ Moderate left to side pleural effusion	Feeding difficulties, mild to moderate learning difficulties, short stature, epilepsy	Never started	-	-	Death due to prolonged seizure resulting in cardiac arrest	None	Gordon et al., 2022 [23]
*RIT1* (NM_006912.5): c.246T>G, p.Phe82Leu (het, d.n.)	Mild SBS	Progressive lower limb and genital lymphoedema, Chylothorax, pericardial effusion with cardiac tamponade, protein-losing enteropathy, ascites/ Reflux of lymphatic fluid into the penoscrotal mass, leakage of contrast bilaterally into the pleural effusions and into the small bowel mesentery	Not reported	22 y	1 mg/day for a month then 2 mg/day	22 mo	Total resolution of ascites in 3 months and of pericardial effusion in 18 months, bettering of the nutritional state	Nausea, gastritis, constipation, eczema of the lower legs, iron deficiency anemia	Gordon et al., 2022 [23]
*RIT1* (NM_006912.5): c.246T>G p.Phe82Leu (het)	Severe HCM with severe left ventricular outflow tract obstruction, SBS, mitral valve dysplasia with moderate stenosis and mild to moderate regurgitation.	Refractory bilateral chylous effusion/ dilated lymphatics along the bilateral iliac vessels.	Not reported	4 y	0.018 mg/kg/day initially every other day for 3 days, then every 36 h for 3 days, then daily	1 y	No progression of HCM, improvement of respiratory function, lymphatic dysplasia and growth.	None	Nakano et al., 2022 [24]
*SOS1* (NM_001382395.1): c.1322G>A, p.Cys441Tyr (het)	Moderate SBS	Ascites, bilateral chylous pleural effusion/ Markedly dilated and malformed lymphatics in the bilateral hila, intercostal spaces, and lungs extending to the left side of neck, to retroperitoneum and pelvis	Esophageal atresia with tracheoesophageal fistula	3 mo	0.026 mg/kg/day for 3 days, then every 36 h for 3 days, then daily	1 y	Resolution of pleural effusion (no recurrence of chylous effusion), improvement of respiratory function, lymphatic dysplasia, SBS and growth	Grade 2 skin irritation	Nakano et al., 2022 [24]
*PTPN11* (NM_001330437): c.854T>C, p.Phe285Ser (het)	Moderate to severe HCM, moderate SBS.	Persistent chylous effusion/ Dilated retroperitoneal and intrathoracic lymphatics with chylolymphatic reflux into the intercostal lymphatics, hila, and pulmonary parenchyma	Myeloproliferative disorder	4 mo	0.023 mg/kg/day for 3 days, then every 36 h for 3 days, and then daily	1 y	Reducing of left ventricular mass, decreased NT-proBNP, slight improvement in the pulmonary valve gradient, bettering of respiratory function, chylous effusion and ascites Death several weeks after discharge for a sudden cardiac event.	None	Nakano et al., 2022 [24]
*SOS1* (NM_005633.4): c.1655G>C, p.Arg552Thr (het)	Small muscular interventricular septal defects, mild SPS/ MAT	Bilateral pleural effusion	Not reported	9 wks	0.02 mg/kg/day	4 mo	Resolution of pleural effusion and MAT (in 72 h), improvement in respiratory function	None	Lioncino et al., 2022 [30]
*RIT1* (NM_006912.5): c.170C >G, p.Ala57Gly (het, d.n.)	HCM, Increased right ventricular outflow tract obstruction, SBS, dysplastic parachute mitral valve	Not reported	Not reported	6 mo	0.025 mg/kg/day, (maximal dosage of 0.04 mg/kg/day)	30 mo	Normalizing of left ventricular wall thickness, decreasing of right ventricular outflow tract obstruction, bettering of growth	Dry and skin rash	Leegaard et al., 2022 [25]
*RIT1* (NM_006912.5): not reported (het)	HCM, left ventricular outflow tract obstruction, severe right ventricular outflow tract obstruction, SBS	Bilateral chylothorax	Bowel perforation	3 y and 4 mo	0.032 mg/kg/day for 3 mo.	11 mo	Reduction in left ventricular mass, no chylothorax recurrence	Eczema of the arms, legs, and trunk Increasing of stoma output	Hribernik et al., 2023 [26]
*RAF1* (NM_002880.4): c.770C>T, p.Ser257Leu (het, d.n.)	HCM, increased LVOT gradient, apical aneurysm, ectatic coronary arteries	Not reported	Obstructive sleep apnea	18 y	0.01 mg/kg	18 mo	Reduction LVOT gradient and N-terminal pro–B-type natriuretic peptide	Not reported	Kiamanesh, et al., 2024 [28]
*PTPN11* (NM_001330437): c.922A>G, p.Asn308Asp (het)	None	Bilateral chylothorax/antegrade retroperitoneal lymph flow and dermal backflow, Hydrops	Not reported	2 wks	0.0125 mg/Kg/day for 2 wks, after 0.0125 mg/kg/day (max dose 0.018 mg/kg/day)	18 mo	Resolution of chylothorax and hydrops	Eczema	Leenders et al., 2024 [27]
*PTPN11* (NM_001330437): c.854T>C, p.Phe285Ser (het)	biventricular HCM, SBS	Right pleural chylous effusion	Ptosis, down-slanted palpebral fissures, marked webbed neck, epicanthic folds, low-set posteriorly rotated ears and prominent frontal bossing	118th day of life	0.025 mg/kg daily	18 mo	Resolution of chylothorax, reduction in left ventricular wall thickness	Transient eczema	Our case report

ABT: age at the beginning of trametinib; Het: heterozygous; d.n.: de novo; HCM: hypertrophic cardiomyopathy; wk(s): week(s); mo: month(s); y: year(s); SBS: sub pulmonary stenosis; DCEMRL: dynamic contrast-enhanced MR lymphangiography; MAT: multifocal atrial tachycardia, LVOT: left ventricular outflow tract.

## Data Availability

Data are contained within the article.

## References

[1] Tartaglia M., Mehler E.L., Goldberg R., Zampino G., Brunner H.G., Kremer H., van der Burgt I., Crosby A.H., Ion A., Jeffery S. (2001). Mutations in PTPN11, encoding the protein tyrosine phosphatase SHP-2, cause Noonan syndrome. Nat. Genet..

[2] Roberts A.E., Allanson J.E., Tartaglia M., Gelb B.D. (2013). Noonan syndrome. Lancet.

[3] Kouz K., Lissewski C., Spranger S., Mitter D., Riess A., Lopez-Gonzalez V., Luttgen S., Aydin H., von Deimling F., Evers C. (2016). Genotype and phenotype in patients with Noonan syndrome and a RIT1 mutation. Genet. Med..

[4] Johnston J.J., van der Smagt J.J., Rosenfeld J.A., Pagnamenta A.T., Alswaid A., Baker E.H., Blair E., Borck G., Brinkmann J., Craigen W. (2018). Autosomal recessive Noonan syndrome associated with biallelic LZTR1 variants. Genet. Med..

[5] van Der Burgt I., Brunner H. (2000). Genetic heterogeneity in Noonan syndrome: Evidence for an autosomal recessive form. Am. J. Med. Genet..

[6] Bhambhani V., Muenke M. (2014). Noonan syndrome. Am. Fam. Physician.

[7] Tartaglia M., Gelb B.D. (2005). Noonan syndrome and related disorders: Genetics and pathogenesis. Annu. Rev. Genom. Hum. Genet..

[8] Jongmans M.C., van der Burgt I., Hoogerbrugge P.M., Noordam K., Yntema H.G., Nillesen W.M., Kuiper R.P., Ligtenberg M.J., van Kessel A.G., van Krieken J.H. (2011). Cancer risk in patients with Noonan syndrome carrying a PTPN11 mutation. Eur. J. Hum. Genet..

[9] Shoji Y., Ida S., Niihori T., Aoki Y., Okamoto N., Etani Y., Kawai M. (2019). Genotype-phenotype correlation analysis in Japanese patients with Noonan syndrome. Endocr. J..

[10] Nakhaei-Rad S., Haghighi F., Bazgir F., Dahlmann J., Busley A.V., Buchholzer M., Kleemann K., Schanzer A., Borchardt A., Hahn A. (2023). Molecular and cellular evidence for the impact of a hypertrophic cardiomyopathy-associated RAF1 variant on the structure and function of contractile machinery in bioartificial cardiac tissues. Commun. Biol..

[11] Gelb B.D., Roberts A.E., Tartaglia M. (2015). Cardiomyopathies in Noonan syndrome and the other RASopathies. Prog. Pediatr. Cardiol..

[12] Yaoita M., Niihori T., Mizuno S., Okamoto N., Hayashi S., Watanabe A., Yokozawa M., Suzumura H., Nakahara A., Nakano Y. (2016). Spectrum of mutations and genotype-phenotype analysis in Noonan syndrome patients with RIT1 mutations. Hum. Genet..

[13] Stuurman K.E., Joosten M., van der Burgt I., Elting M., Yntema H.G., Meijers-Heijboer H., Rinne T. (2019). Prenatal ultrasound findings of rasopathies in a cohort of 424 fetuses: Update on genetic testing in the NGS era. J. Med. Genet..

[14] Sleutjes J., Kleimeier L., Leenders E., Klein W., Draaisma J. (2022). Lymphatic Abnormalities in Noonan Syndrome Spectrum Disorders: A Systematic Review. Mol. Syndromol..

[15] Witt D.R., Hoyme H.E., Zonana J., Manchester D.K., Fryns J.P., Stevenson J.G., Curry C.J., Hall J.G. (1987). Lymphedema in Noonan syndrome: Clues to pathogenesis and prenatal diagnosis and review of the literature. Am. J. Med. Genet..

[16] Joyce S., Gordon K., Brice G., Ostergaard P., Nagaraja R., Short J., Moore S., Mortimer P., Mansour S. (2016). The lymphatic phenotype in Noonan and Cardiofaciocutaneous syndrome. Eur. J. Hum. Genet..

[17] Swarts J.W., Kleimeier L.E.R., Leenders E., Rinne T., Klein W.M., Draaisma J.M.T. (2022). Lymphatic anomalies during lifetime in patients with Noonan syndrome: Retrospective cohort study. Am. J. Med. Genet. A.

[18] Hoffner B., Benchich K. (2018). Trametinib: A Targeted Therapy in Metastatic Melanoma. J. Adv. Pract. Oncol..

[19] O’Leary C.G., Andelkovic V., Ladwa R., Pavlakis N., Zhou C., Hirsch F., Richard D., O’Byrne K. (2019). Targeting BRAF mutations in non-small cell lung cancer. Transl. Lung Cancer Res..

[20] Andelfinger G., Marquis C., Raboisson M.J., Theoret Y., Waldmuller S., Wiegand G., Gelb B.D., Zenker M., Delrue M.A., Hofbeck M. (2019). Hypertrophic Cardiomyopathy in Noonan Syndrome Treated by MEK-Inhibition. J. Am. Coll. Cardiol..

[21] Dori Y., Smith C., Pinto E., Snyder K., March M.E., Hakonarson H., Belasco J. (2020). Severe Lymphatic Disorder Resolved with MEK Inhibition in a Patient with Noonan Syndrome and SOS1 Mutation. Pediatrics.

[22] Mussa A., Carli D., Giorgio E., Villar A.M., Cardaropoli S., Carbonara C., Campagnoli M.F., Galletto P., Palumbo M., Olivieri S. (2021). MEK Inhibition in a Newborn with RAF1-Associated Noonan Syndrome Ameliorates Hypertrophic Cardiomyopathy but Is Insufficient to Revert Pulmonary Vascular Disease. Genes.

[23] Gordon K., Moore M., Van Zanten M., Pearce J., Itkin M., Madden B., Ratnam L., Mortimer P.S., Nagaraja R., Mansour S. (2022). Case Report: Progressive central conducting lymphatic abnormalities in the RASopathies. Two case reports, including successful treatment by MEK inhibition. Front. Genet..

[24] Nakano T.A., Rankin A.W., Annam A., Kulungowski A.M., McCallen L.M., Hill L.R., Chatfield K.C. (2022). Trametinib for Refractory Chylous Effusions and Systemic Complications in Children with Noonan Syndrome. J. Pediatr..

[25] Leegaard A., Gregersen P.A., Nielsen T.O., Bjerre J.V., Handrup M.M. (2022). Succesful MEK-inhibition of severe hypertrophic cardiomyopathy in RIT1-related Noonan Syndrome. Eur. J. Med. Genet..

[26] Hribernik I., Brooks T., Dunlop-Jones A., Bentham J.R. (2023). Successful treatment of refractory chylothorax with MEK inhibitor trametinib in a child with Noonan syndrome: Case report. Eur. Heart J. Case Rep..

[27] Leenders E., Kleimeier L.E.R., Weeke L.C., Coppens C.H., Klein W.M., Draaisma J.M.T. (2024). Trametinib restores the central conducting lymphatic flow in a premature infant with Noonan syndrome. Clin. Case Rep..

[28] Kiamanesh O., Greenway S.C., Dicke F., Ballantyne B., Mitrovic S., McGrath K., White J.A., Kent W.D.T., Andelfinger G. (2024). Treatment of RAF1-Related Obstructive Hypertrophic Cardiomyopathy by MEK Inhibition Using Trametinib. JACC Case Rep..

[29] Meisner J.K., Bradley D.J., Russell M.W. (2021). Molecular Management of Multifocal Atrial Tachycardia in Noonan’s Syndrome with MEK1/2 Inhibitor Trametinib. Circ. Genom. Precis. Med..

[30] Lioncino M., Fusco A., Monda E., Colonna D., Sibilio M., Caiazza M., Magri D., Borrelli A.C., D’Onofrio B., Mazzella M.L. (2022). Severe Lymphatic Disorder and Multifocal Atrial Tachycardia Treated with Trametinib in a Patient with Noonan Syndrome and SOS1 Mutation. Genes.

[31] Richards S., Aziz N., Bale S., Bick D., Das S., Gastier-Foster J., Grody W.W., Hegde M., Lyon E., Spector E. (2015). Standards and guidelines for the interpretation of sequence variants: A joint consensus recommendation of the American College of Medical Genetics and Genomics and the Association for Molecular Pathology. Genet. Med..

[32] Gelb B.D., Yohe M.E., Wolf C., Andelfinger G. (2022). New prospectives on treatment opportunities in RASopathies. Am. J. Med. Genet. C Semin. Med. Genet..

[33] Soto-Martinez M., Massie J. (2009). Chylothorax: Diagnosis and management in children. Paediatr. Respir. Rev..

[34] Agarwal S., Anderson B.K., Mahajan P., Fernandes C.J., Margolin J.F., Iacobas I. (2022). Sirolimus efficacy in the treatment of critically ill infants with congenital primary chylous effusions. Pediatr. Blood Cancer.

[35] Tamaoka S., Osada A., Kin T., Arimitsu T., Hida M. (2021). Midodrine, an Oral Alpha-1 Adrenoreceptor Agonist, Successfully Treated Refractory Congenital Chylous Pleural Effusion and Ascites in a Neonate. Chest.

[36] Hebron K.E., Hernandez E.R., Yohe M.E. (2022). The RASopathies: From pathogenetics to therapeutics. Dis. Models Mech..

[37] Casey D., Demko S., Sinha A., Mishra-Kalyani P.S., Shen Y.L., Khasar S., Goheer M.A., Helms W.S., Pan L., Xu Y. (2021). FDA Approval Summary: Selumetinib for Plexiform Neurofibroma. Clin. Cancer Res..

[38] Perreault S., Larouche V., Tabori U., Hawkin C., Lippe S., Ellezam B., Decarie J.C., Theoret Y., Metras M.E., Sultan S. (2019). A phase 2 study of trametinib for patients with pediatric glioma or plexiform neurofibroma with refractory tumor and activation of the MAPK/ERK pathway: TRAM-01. BMC Cancer.

[39] Infante J.R., Fecher L.A., Falchook G.S., Nallapareddy S., Gordon M.S., Becerra C., DeMarini D.J., Cox D.S., Xu Y., Morris S.R. (2012). Safety, pharmacokinetic, pharmacodynamic, and efficacy data for the oral MEK inhibitor trametinib: A phase 1 dose-escalation trial. Lancet Oncol..

[40] Zhou L., Feng Y., Ma Y.C., Zhang Z., Wu J.W., Du S., Li W.Y., Lu X.H., Ma Y., Wang R.L. (2021). Exploring the mechanism of the potent allosteric inhibitor compound2 on SHP2 (WT) and SHP2(F285S) by molecular dynamics study. J. Mol. Graph. Model..

[41] Watanabe D., Hasebe Y., Kasai S., Shinohara T., Maebayashi Y., Katsumata N., Nemoto A., Naitoh A. (2022). PTPN11 c.853T>C (p.Phe285Leu) mutation in Noonan syndrome with chylothorax. Nagoya J. Med. Sci..

[42] Kondyli M., Larouche V., Saint-Martin C., Ellezam B., Pouliot L., Sinnett D., Legault G., Crevier L., Weil A., Farmer J.P. (2018). Trametinib for progressive pediatric low-grade gliomas. J. Neurooncol..

[43] Bouffet E., Geoerger B., Moertel C., Whitlock J.A., Aerts I., Hargrave D., Osterloh L., Tan E., Choi J., Russo M. (2023). Efficacy and Safety of Trametinib Monotherapy or in Combination with Dabrafenib in Pediatric BRAF V600-Mutant Low-Grade Glioma. J. Clin. Oncol..

[44] Paul M.R., Pehlivan K.C., Milburn M., Yeh-Nayre L., Elster J., Crawford J.R. (2020). Trametinib-based Treatment of Pediatric CNS Tumors: A Single Institutional Experience. J. Pediatr. Hematol. Oncol..

